# Variability and Reproducibility of Directed and Undirected Functional MRI Connectomes in the Human Brain

**DOI:** 10.3390/e21070661

**Published:** 2019-07-06

**Authors:** Allegra Conti, Andrea Duggento, Maria Guerrisi, Luca Passamonti, Iole Indovina, Nicola Toschi

**Affiliations:** 1 Laboratory of Neuromotor Physiology, IRCCS Santa Lucia Foundation, 00179 Rome, Italy; 2 Department of Biomedicine and Prevention, University of Rome Tor Vergata, 00133 Rome, Italy; 3 Department of Clinical Neurosciences, University of Cambridge, Cambridge CB2 0QQ, UK; 4 Institute of Bioimaging and Molecular Physiology, National Research Council, 20090 Milano, Italy; 5 Saint Camillus International University of Health and Medical Sciences, 00131 Rome, Italy; 6 Department of Radiology, Athinoula A. Martinos Center for Biomedical Imaging, Boston, MA 02129, USA

**Keywords:** functional networks, functional magnetic resonance imaging, connectome, connectivity matrices, graphs, reproducibility, granger causality, transfer entropy

## Abstract

A growing number of studies are focusing on methods to estimate and analyze the functional connectome of the human brain. Graph theoretical measures are commonly employed to interpret and synthesize complex network-related information. While resting state functional MRI (rsfMRI) is often employed in this context, it is known to exhibit poor reproducibility, a key factor which is commonly neglected in typical cohort studies using connectomics-related measures as biomarkers. We aimed to fill this gap by analyzing and comparing the inter- and intra-subject variability of connectivity matrices, as well as graph-theoretical measures, in a large (n = 1003) database of young healthy subjects which underwent four consecutive rsfMRI sessions. We analyzed both directed (Granger Causality and Transfer Entropy) and undirected (Pearson Correlation and Partial Correlation) time-series association measures and related global and local graph-theoretical measures. While matrix weights exhibit a higher reproducibility in undirected, as opposed to directed, methods, this difference disappears when looking at global graph metrics and, in turn, exhibits strong regional dependence in local graphs metrics. Our results warrant caution in the interpretation of connectivity studies, and serve as a benchmark for future investigations by providing quantitative estimates for the inter- and intra-subject variabilities in both directed and undirected connectomic measures.

## 1. Introduction

The interest in studying directed and undirected interactions between different regions in the human brain (i.e., the functional ‘connectome’) is growing exponentially [1,2,3,4,5,6,7], and the advent of graph-theoretical applications to neuroscience has provided additional avenues to represent, analyze and interpret information contained in complex, possibly dynamic networks like the human connectome [8,9,10,11,12]. Functional connectome estimates are typically derived from neuromonitoring data (e.g., resting state functional MRI—rsfMRI), and established methods for computing whole-brain functional connectivity matrices include both undirected and directed estimators [13,14,15]. As is well known, the reproducibility of MRI data varies widely across modalities, and rsfMRI data are known to exhibit significant inter- and intra-subject fluctuations [16,17,18]. While this can significantly bias and hamper the interpretation of functional connectivity studies, which do not typically include targeted scan-rescan experiments to assess reproducibility, the impact of this variability on connectome matrices and related measures has not yet been systematically investigated. 

In this study, we explore, quantify and compare the intra- and inter-subject variability (and hence reproducibility) of both directed and undirected resting state functional connectivity measures in a large (1003 subjects) database of high-quality rsfMRI data consisting of 4 scan sessions per subject. In detail, we investigate the reproducibility of whole-brain adjacency matrices derived from Pearson Correlation (PearC), Partial Correlation (PartC) as well as multivariate Granger Causality (mGC) and multivariate Transfer Entropy (mTE). In addition, we investigate the reproducibility of both global (whole-brain) and local (node-wise) graphs metrics calculated for all four types of adjacency matrices.

While PearC and PartC estimate zero-lag, non-directional associations between time-series, mGC and mTE inherently measure directed information transfer. Such directed measures have already been successfully employed in brain connectivity studies [5,19,20,21,22,23] and, while it is known that mTE and mGC are equivalent in the limit of Gaussian data [24], these two estimators may differ when applied when the data distribution is skewed and/or the number of data points is extremely limited. mGC is based on choice of model parameters [25], while TE is model-free and possibly a superior tool for estimating nonlinear interactions [15] like the ones commonly found in biological signals.

## 2. Materials and Methods 

### 2.1. rsfMRI Data

We employed rsfMRI data from 1003 subjects, part of the Human Connectome Project (HCP) (S1200 PTN release [26]). Each subject underwent a total of 4 resting state scans (2 sessions on 2 different days, 2 scans per session, 1200 timepoints/scan, TR 720 ms, TE 33.1 ms, flip angle 52, FOV 208 × 180, thickness 2.0 mm; 72 slices; 2.0 mm isotropic voxel size, multiband factor 8, echo spacing 0.58 ms, BW 2290 Hz/Px, 14 minutes of acquisition time/scan). Subjects were scanned on a customized Siemens Skyra 3 T scanner [26] (Smith et al. 2013) with higher spatial and temporal resolutions as compared to common fMRI acquisition protocols. Data pre-processing was state-of-the-art and included gradient distortion and motion correction, field map pre-processing using spin echo field map (specific for each scanning day), intensity normalization and bias field removal. Residual artifacts were removed through an automatic classifier specifically trained to this particular dataset, which is able to remove measurement noise, additional motion or physiological artifacts with extremely high accuracy. Further details regarding data processing can be found in the HCP S1200 Release reference manual [https://www.humanconnectome.org/storage/app/media/documentation/s1200/HCP_S1200_Release_Reference_Manual.pdf].

**Figure 1:**
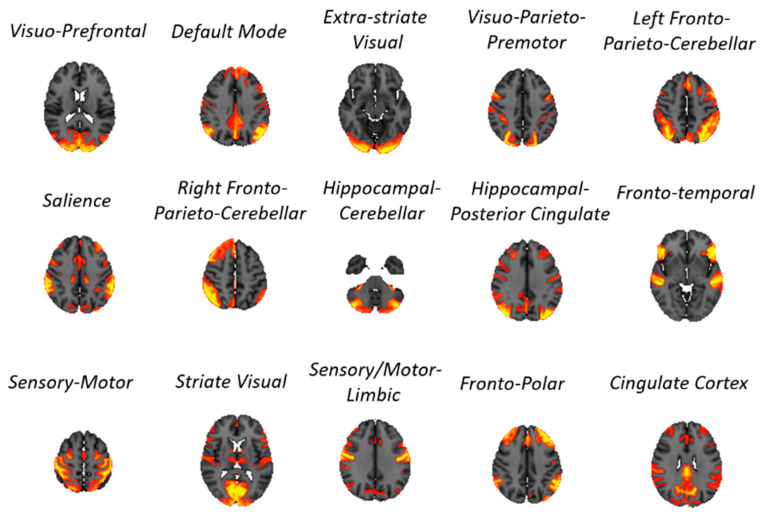
Brain functional subnetworks (i.e., independent components) identified by group independent component analysis (gICA) in 1003 subjects drawn from the Human Connectome Project database. The subject-wise 15-node specific time-series relative to these components were employed for all analyses in this paper.

In this paper, we processed subject- and scan-wise fMRI timeseries (1200 points each) resulting from group independent component analysis (gICA) at dimensionality 15 (made available by the HCP consortium). Exemplary slices of the resulting node (i.e., component, also called “subnetworks” in this paper) maps, along with their physiological interpretations [27], are shown in Figure 1. To cater for confounding elements introduced by locally varying hemodynamic response functions (HRFs) [28], fMRI timeseries were preprocessed using a the blind deconvolution approach [29] (maximum lag 10 s = 14 time points, threshold: 1 standard deviation) implemented in a publicly available toolbox (https://users.ugent.be/~dmarinaz/HRF_deconvolution.html). Stationarity of all signals was verified using the Augmented Dickey-Fuller test. All signals were successively standardized prior to additional processing.

### 2.2. Estimation of Adjacency Matrices

**Figure 2:**
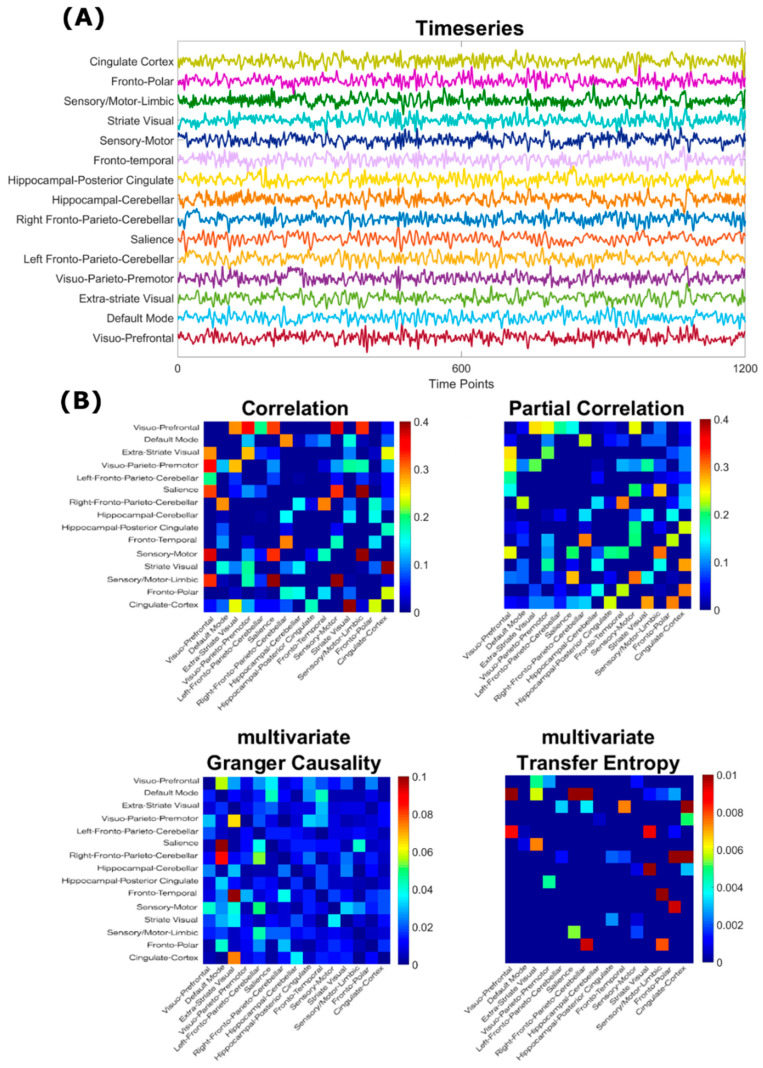
(**A**) Example node-wise timeseries from a single scan, used to compute subject-, session- and scan-specific adjacency matrices; (**B**) Examples of adjacency matrices obtained from both undirected (first row) and directed (second row) connectivity estimators for one subject. For each method, the median across 4 rsfMRI sessions from a single subject is shown. Diagonal elements are set to zero.

Starting from the 15 subject-, scan- and node-specific timeseries (see Figure 2A for an example), subject-wise undirected adjacency matrices were obtained through both PearC and PartC between all pairs of timeseries through in-house code written in in MATLAB (v. 2018a). Negative correlations were set to zero. Directed adjacency matrices where obtained through mGC estimates, in its most recent state-space formulation [30,31], and through mTE.

mGC and mTE were calculated through in-house modified versions of the Matlab Tools for the computation of multiscale Granger Causality (http://www.lucafaes.net/msGC.html) [31] and of multivariate Transfer Entropy (http://www.lucafaes.net/cTE.html) [32]. In the case of GC, the autoregressive order was chosen my minimizing the median Schwartz criterion across all subjects (order = 5). In mTE, conditioning vectors are formed according to a non-uniform building scheme selecting past terms up to a maximum lag of 5 time points while minimizing the conditional entropy [32]. Examples of adjacency matrices (which are symmetrical for undirected estimators, and asymmetrical for directed ones) obtained through all four methods are shown in Figure 2B.

### 2.3. Global and Local Graph Metric Estimation

Starting from the adjacency matrices, we computed both global and local graph-theoretical indices of functional connectivity for all three methods/estimators. All graph measures were computed via the Brain Connectivity Toolbox [33] (https://sites.google.com/site/bctnet/), by using functions available for weighted adjacency matrices for both directed (in case of mGC and mTE) and undirected (for pearC and partC) graph metrics.

**Figure 3:**
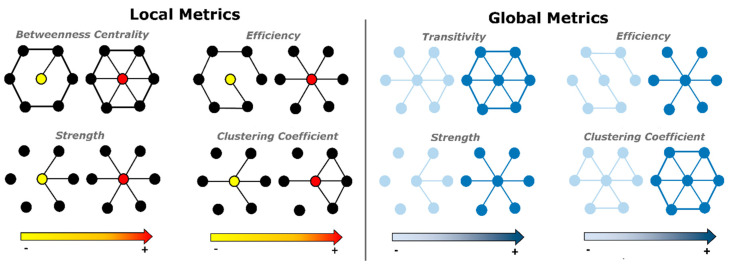
Exemplification of local (**left**) and global (**right**) graph metrics as a function of node neighborhood connectivity. Local metrics (**left**): taking the yellow/red center node as a reference, its betweenness centrality and local efficiency increase as the average shortest path between the center node and all its neighbors decreases. Strength/degree is proportional to the number of connections, the clustering coefficient increases with the ratio between the number of links actually present between the vertices within node neighborhood and the number of links that could possibly exist between them. Global metrics (**right**): transitivity and clustering coefficient both reflect prevalence of clustered connectivity around nodes, while efficiency and strength increase with the increase of the local efficiency and strength of each node in the graph [33].

For each subject and for each scan, we calculated graph metrics quantifying the centrality of a node within a network (local strength and betweenness centrality), its ability to transmit information at local level (local efficiency) and its integration properties (clustering coefficient) [33]. Also, since global graph metrics characterize the overall organization of a network, we computed the global strength, the global efficiency and the global clustering coefficient as the average of the respective local metrics of all nodes. In addition, we calculated graph transitivity (a property related to the existence of tightly connected communities of nodes). Figure 3 exemplifies the meaning and calculation of these graph-theoretical metrics.

### 2.4. Inter- and Intra-Subject Variability Distributions

Adjacency matrix weights (both directed and undirected) and both local and global graph-theoretical measures, have their own native scales and dimensionalities and, in the case of local graph metrics, this scale also depends on the specific brain region. The same issue, which results in dimensional and extremely different effect sizes across estimators, also affects graph-theoretical metrics calculated from different estimators. Therefore, in order to compare inter- and intra-subject fluctuations in adjacency matrices, as well as graph-theoretical metrics, we employed dimensionless, normalized pairwise quantifiers of asymmetry, along with their estimated distributions.

In detail, we defined the pairwise normalized difference/asymmetry (ND) as:

(1) $$\mathrm{ND}=\frac{a-b}{a+b}$$

where *a* and *b* refer to two distinct scans (either intra- or inter-subject). ND is both normalized (i.e., a-dimensional) and bounded (i.e., between −1 and 1).

In the case of scalar quantities like global or local graph-theoretical metrics, Equation (1) can be applied directly. When comparing adjacency matrices, Equation (1) was modified by using Frobenius distances between (in this case) matrices *a* and *b* as follows:

(2) $$\mathrm{ND}=\sqrt{\frac{trace\left(\left(a-b\right)\cdot {\left(a-b\right)}^{\prime }\right)}{trace\left(\left(a+b\right)\cdot {\left(a+b\right)}^{\prime }\right)}}$$

**Figure 4:**
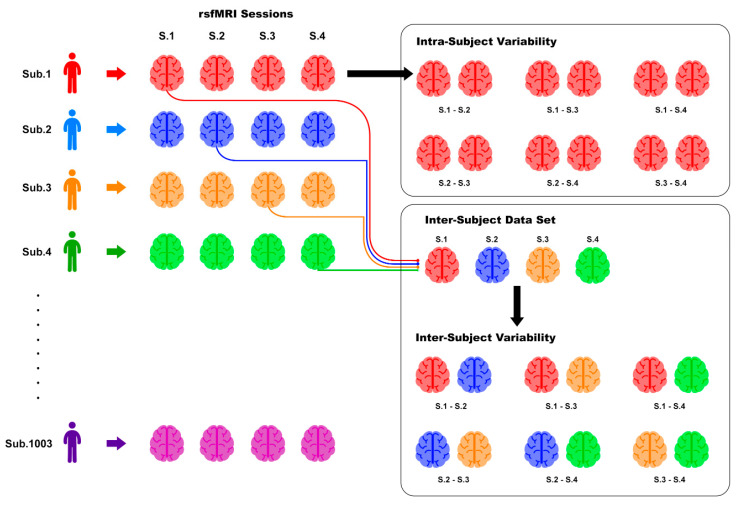
Sampling strategy used to construct intra- and inter-subject pairs and subsequent distributions of ND (Equations (1) and (2)). The constructed samples for the basis for computing the distributions are shown, e.g., in Figure 5 and Figure 6.

To empirically estimate (and successively compare) the intra- and inter-subject distributions in ND, we proceeded as follows. From each set of 4 subject-wise scans, we sampled all 6 possible pairs of scans, computed the ND for each pair, and repeated this step for all 1003 subjects. For each method, this resulted in 1003 × 6 = 6018 distinct ND values referring to intra-subject variability. Subsequently, in order to build a comparable distribution related to inter-subject variability, we randomly and repeatedly sampled (without replacement) four scans from four distinct subjects, hence constructing set of 4 “inter-subject” scans from which 6 distinct pairs could be constructed and 6 distinct ND values could be calculated as above. To control for possible differences arising from intra-subject habituation, the random sampling was performed so that each “inter-subject” set of four scans contained exactly 1 sample from the 1003-sized set of first, second, third and fourth scans. This procedure was repeated 1003 times, resulting in 1003 × 6 = 6018 distinct ND values referring to inter-subject variability. Figure 4 summarizes the sampling process.

### 2.5. Statistical Analysis

After sampling and computation of all ND values for each graph-theoretical metric and for all four types of adjacency matrix, the resulting “inter” and “intra” distribution medians of ND were compared through nonparametric Mann-Whitney-U tests. Effect Size (ES) were evaluated as the difference between the medians of the “inter” and “intra” distributions in ND (i.e., ES = “inter” − “intra”). The result of each comparison is therefore a *p*-value with an associated ES. Additionally, in order to explore possible differences in variability across nodes (i.e., brain regions), we pooled ND values for all local graph-theoretical metrics across all four methods (4 metrics × four methods = 16 values per node), and tested the effect of anatomical localization using a nonparametric Kruskal-Wallis test. Whenever a significant effect was found, pairwise comparison between nodes was performed in order to further examine which pairwise differences were the main drivers of the overall effect. This procedure was repeated separately for inter- and intra-subject variability.

## 3. Results

**Figure 5:**
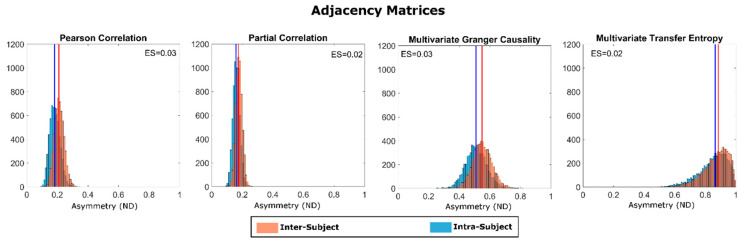
Intra- (blue) and inter- (orange) subject distributions of the Normalized Differences (ND), for all four connectivity estimation methods. Effect size (ES) (i.e., the difference between the median values of the inter- and intra-subject ND distributions) are shown in the insets. Mann-Whitney U-tests for comparing medians returned *p* < 10^−20^ for all four methods.

Figure 5 shows the “inter” and “intra” distribution for ND obtained when comparing pairs of adjacency matrices (Equation (2)) for all four methods (PearC, PartC, mGC and mTE), along with effect sizes. For all methods, as hypothesized, median intra-subject variability is significantly lower than median inter subject variability (*p* < 10^−20^ for all methods). The largest ES was associated with mGC and PearC (ES = 0.03 for both), followed by PartC and mTE (ES = 0.02 for both). Qualitatively, mTE showed the largest median variabilities (both intra- and inter-subject), whereas PearC and PartC showed similar median variabilities. 

**Figure 6:**
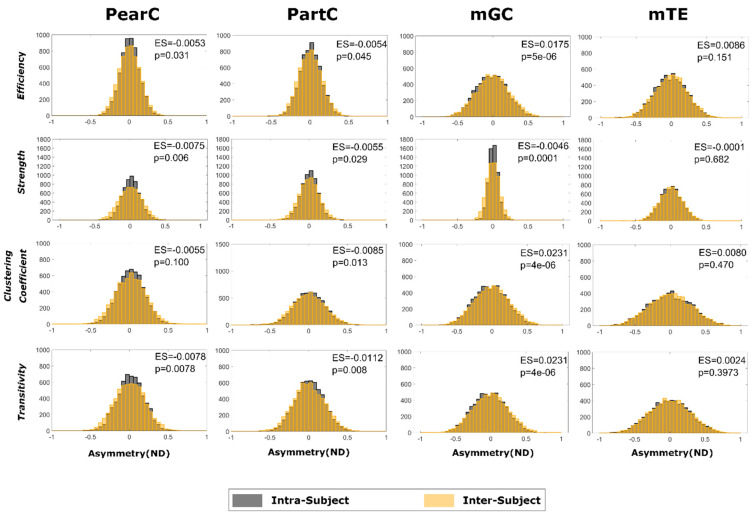
Intra- (gray) and inter- (orange) subject distributions of the Normalized Differences (ND), for all four connectivity estimation methods, along with the *p*-value resulting from the corresponding Mann-Whitney-U test. Effect size (ES) (i.e., the difference between the median values of the inter- and intra-subject ND distributions) are shown in the insets.

**Figure A1:**
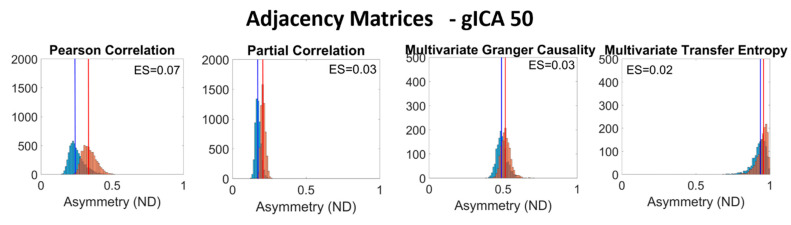
Intra- (blue) and inter- (orange) subject distributions of the Normalized Differences (ND), for adjacency matrices obtained from the ICA decomposition with dimensionality of 50, for all four connectivity estimation methods. Effect size (ES) (i.e., the difference between the median values of the inter- and intra- subject ND distributions) are shown in the insets. Mann-Whitney U-tests for comparing medians returned *p* < 10^−20^ for all four methods.

**Figure A2:**
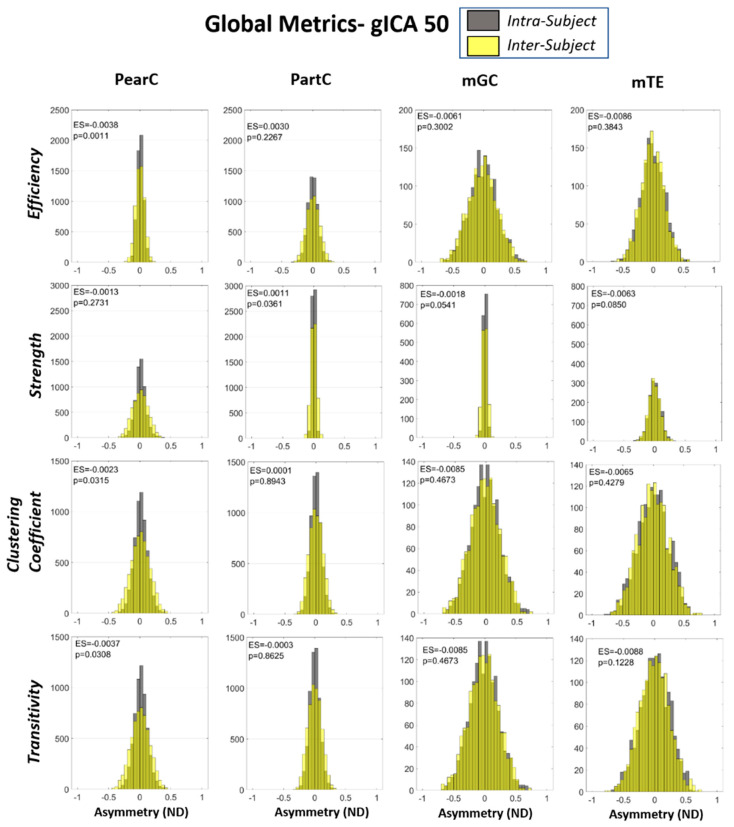
Intra- (black) and inter- (yellow) subject distributions of the Normalized Differences (ND), for all four connectivity estimation methods, using ICA-derived time-series at dimensionality 50. Effect size (ES) (i.e., the difference between the median values of the inter- and intra- subject ND distributions) together with the *p*-values resulting from the Mann-Whitney-U tests, are shown in the insets.

Figure 6 shows the “inter” and “intra” distribution for ND (Equation (1)) obtained when comparing pairs of global graph-theoretical metrics, along with the results of statistical testing in terms of *p*-values and ES. Interestingly, intra- and inter-subject fluctuations are distinguishable only in some cases. In particular, we found statistically significant differences (*p* < 0.05) for all metrics estimated through PearC (named “Bivariate Correlation” in figure) and PartC. However, in these cases, intra-subject fluctuations were larger than inter-subject fluctuations (i.e., negative ES). On the other hand, when looking global metrics calculated through mGC, significant differences between intra- vs. inter-subject fluctuations were mainly associated with positive ES. No differences were found between ND distributions for global-graphs metrics evaluated through mTE. Notably, in this analysis, all significant differences were associated with minimal (compared to unity) effect sizes (of the order of between 10^−2^ and 10^−3^). In addition, the widths of intra- and inter-subject variability distributions were qualitatively similar for all methods. The main analyses presented in Figure 5 and Figure 6 were also repeated using a finer parcellation (50 subnetworks—see supplementary materials (See Figure A1 and Figure A2)). The results were fully compatible (both in effect sizes and directions) with the main results obtained using 15 subnetworks.

**Figure 7:**
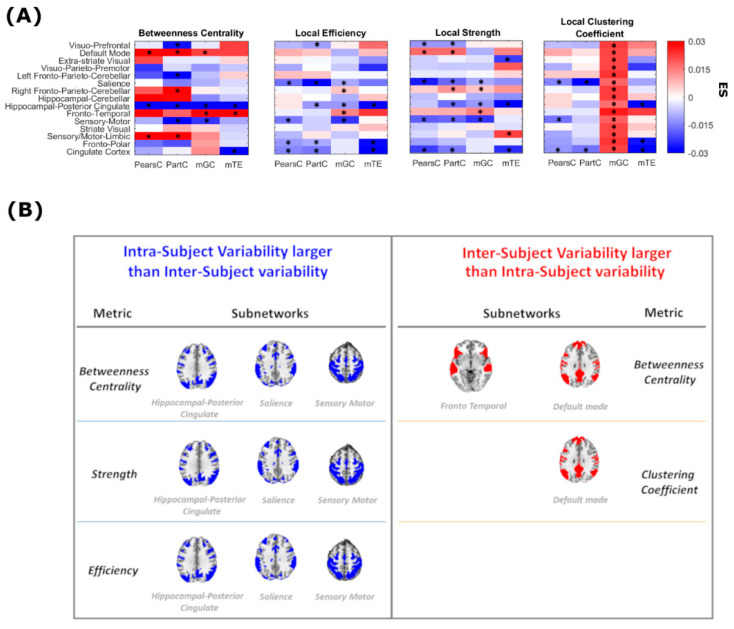
(**A**) Effect size, i.e., differences between median intra- and inter-subject distributions in local graph metrics for each node and for each connectivity estimation method (PearC, PartC, mGC and mTE). For each metric, darker red and blue cells correspond to higher and lower ES values, respectively. The nodes in which differences between inter- and intra-subject variabilities are statistically different from 0 (*p* < 0.05) are indicated with an asterisk; (**B**) Subnetworks (i.e., components) with larger intra-subject than inter-subject variability in various metrics are shown in blue, while subnetworks showing a larger intra-subject reproducibility are shown in red.

Figure 7 summarizes the results of comparing ND distributions computed from local graph-theoretical metrics, along with the results of statistical testing as well as ES. In this case, an intricate, anatomically dependent pattern emerged, where the statistical difference between median intra- and inter-subject fluctuations depends on the specific node, with some nodes exhibiting higher intra-subject (as compared to inter-subject) fluctuations. Asterisks indicate nodes in which differences between inter- and intra-subject variabilities resulted in be different from 0 (*p* < 0.05, Mann-Whitney U test). These results highlight the strong spatial dependence across the brain in reproducibility of all local graph-theoretical indices. Only the betweenness centrality of the Hippocampal-Posterior Cingulate subnetwork show statistically higher intra-subject (as opposed to inter-subject) variability across all methods. Still, ES in local efficiency and strength estimated in the same subnetwork tend to the same direction in all methods. Higher intra-subject (as opposed to inter-subject) variability of the betweenness centrality and local efficiency and strength was also found for the salience and the sensory motor subnetworks. On the other hand, the default mode subnetwork displayed higher intra-subject (as opposed to inter-subject) stability in both betweenness centrality and local clustering coefficient across methods. The same hold for the betweenness centrality of the fronto-temporal subnetwork. The results shown in Figure 7 allow also to qualitatively compare the variability of the local metrics obtained though undirected (PearC and PartC) and directed (mGC and mTE) methods. In particular, local efficiency, strength and clustering coefficient in the visuo-prefrontal subnetwork are highly reproducible within the same subject when estimated using mGC and mTE.

With reference to Figure 7, Table 1 summarizes the number of nodes where statistically significant intra- vs. inter-subject differences were found. Overall, across all four methods (PearC, PartC, mGC and mTE) the percentage of nodes (out of 15) where inter- and intra-subject variability were statistically different varied between 28% and 38% (bottom row of Table 1), and in 3–35% of the 15 nodes, the inter-subject variability is significantly higher than the intra-subject variability. mGC is the method with the lowest average number of nodes (across metrics) where the above-mentioned difference is significantly different from zero. Also, node strength is the metric where the above-mentioned difference is significantly different from zero in the highest average number of nodes (across methods). Importantly, in the absence of a ground-truth, it is not possible to determine which source of variability dominates the overall effects. This could only be ascertained with synthetic data which accurately models neuronal spiking and cardiovascular coupling [9].

**Figure 8:**
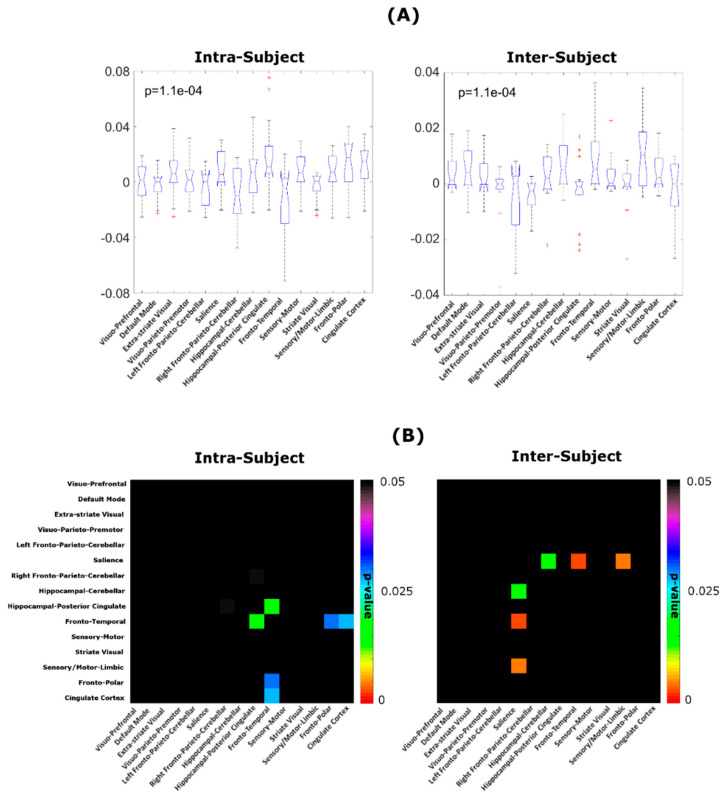
(**A**) Intra and inter ND values (across 1003 subjects; left: intra-subject variability, right: inter-subject variability) for all local metrics (**B**) *p*-values obtained from pairwise comparison of node-specific, intra- and inter-subject variability.

Figure 8 shows the results the Kruskal-Wallis test performed separately on the “intra” and on the “inter” ND values. In both intra- and inter-subject variability we found a significant effect of node (*p* = 1.1 × 10^−4^). In post-hoc comparisons (Figure 8B) we found that overall intra-subject variability in local graph-theoretical metrics of the fronto-temporal subnetwork is lower as compared to the hippocampal-posterior cingulate, the cingulate cortex and the fronto-polar subnetworks. Also, overall inter-subject variability in local graph-theoretical metric is lower in the salience subnetwork as compared to the hippocampal-cerebellar, fronto-temporal and sensory/motor-limbic subnetworks.

## 4. Discussion 

In this paper, we have studied the inter- and intra-subject variability of directed and undirected connectivity matrices as well as of global as well as local graph-theoretical metrics derived from resting state functional MRI data in a large sample (n = 1003) of resting state fMRI data, which also has the unique property of including scan-rescan sessions. While previous reproducibility studies exist, they are commonly focused on specific data processing [34] or statistical [35] aspects, and are usually based on limited sample sizes. 

We employed a fully data-driven parcellation, derived from group independent component analysis performed by the HCP consortium and which is hence independent from any anatomical priors commonly present in classical parcellations. To further eliminate any unwanted sources of variability and improve the comparability between distributions, we adopted a bootstrap approach followed by nonparametric statistics on a normalized difference metric, which also allowed us to pool across methods and/or nodes and make qualitative inter-method comparisons. In addition, in order to investigate the dependence of our results on ICA dimensionality (and hence coarseness/fineness of the resulting parcellation), we repeated the main analyses using data based on a dimensionality of 50 instead of 15. Interestingly, all effect direction and sizes are highly comparable to the main results ad dimensionality 15.

Our results show that while adjacency matrix weights generated using conventional methods like PearC or PartC exhibit 1) a qualitatively lower variability as compared to directed metrics and 2) a lower intra-subject vs. inter-subject variability; these differences virtually disappear when looking at global and local graphs-metrics values, where re-scanning the same subject results in the same statistical fluctuation as scanning a different subject. The overall higher variability in adjacency matrices obtained from directed metrics may be partially due to the higher complexity of both estimators and the multiple degrees of freedom (e.g., model order, embedding dimensions) in their computation, whose exploration was beyond the scope of this study. Also, the appropriateness of using of mGC in fMRI has been the object of discussion [36,37,38,39,40,41,42], mainly because of the confounding elements introduced by locally varying neurovascular coupling and the interaction of these confounds with, e.g., parameter choices, and hence the overall validity of the estimators. To this end, we applied a blind deconvolution approach that estimates a local HRF for each signal and returns an estimate of neural activity. Still, the application of this type of pipeline is not yet widespread in fMRI studies using causality methods, and that it also involves parameter choices whose effect on reproducibility merit investigation in a separate paper. Similarly, while partial and Pearson correlation do not involve parameter optimization, both mGC and mTE estimations are based on parameter choices inherent to each method. In this sense, the main aim of our work was to not to directly compare across methods, but rather to provide an estimation, in a high-quality and unique data sample, of overall the intra- and inter-subject reproducibility of a variety of connectomic metrics which are commonly used in the fMRI community.

Interestingly, we found an articulate pattern of “intra” vs. “inter” variability differences as a function of metrics, as well as node (i.e., anatomical localization), which points towards a certain degree of unpredictability in the reproducibility of results obtained using each possible composite pipeline. Interestingly, in several nodes and metrics, median intra-subject variability was found to be larger than median inter-subject variability. In this context, previous studies have highlighted the higher intra-subject (as compared to inter-subject) variability of functional connectivity in specific regions of the brain [43,44,45,46]. Indeed, intra-subject variability depends on several factors such as diurnal rhythms [47,48], cognitive/behavioral contexts [49,50,51], caffeine intake [43], sleepiness [52,53,54] and attention [46,55], which may explain the high inter-subject fluctuations we found in this paper.

### Limitations and Future Perspectives

We employed public HCP time-series data stemming from a group ICA at dimensionality 15. Several previous investigations have extracted useful information employing “small” networks and graph metrics [10,20,56,57]. Also, additional analyses performed at higher dimensionality confirm the main results of this paper. However, we cannot exclude that the fluctuations quantified in this study may change as a function of parcellation, and should therefore be investigated ad hoc on the basis of data specifications and parcellation details in any specific study where a reproducibility assessment is warranted, e.g., in longitudinal investigations. Also, in view of the advent of novel connectivity estimators, like, e.g., Generalized Recurrent Neural Network-based Dynamic Causal Modeling [58] or model-based whole-brain effective connectivity (MOU-EC) [59], future investigations are needed to characterize the repeatability and robustness of the connectivity matrices generated by such strategies. In addition, since dynamical connectivity measures and connectivity density are also likely to be impacted by reproducibility issues [60], a study of the robustness of dynamic functional connectomes, based on nonparametric distribution-building approaches like the one we employed in this paper, appears warranted. Finally, future investigation of the reproducibility of the regional dependence of functional connectivity by using large-scale brain network models would also be useful for better understanding the causes of intra- and inter-subject fluctuations in conventional connectivity estimators we observed in this study.

## 5. Conclusions

In this paper, we have studied inter- and intra-subject variability of directed and undirected connectivity matrices, as well as of global and local graph-theoretical metrics derived from resting state functional MRI data in a large sample (n = 1003) of resting state fMRI data released by the HCP. We analyzed both directed (Granger Causality and Transfer Entropy) and undirected (Pearson Correlation and Partial Correlation) association measures and derived graph-theoretical measures.

Our results show that adjacency matrices exhibit a higher intra-subject (as compared to inter-subject) reproducibility. However, intra- and inter-subject dispersion of both global and local graphs-metrics resulted as being comparable. We therefore demonstrate that, even with large volumes of densely sampled rsfMRI data, reproducibility is an issue that should be constantly kept in mind and that connectomics studies should, whenever possible, include a scan-rescan validation arm, at least in a subset of subjects. Our results also serve as benchmarks for future investigations aiming to estimate the variability in other directed and undirected connectomic databases, and can also be employed for prospective power calculations in planning functional connectomics experiments.

## Figures and Tables

**Table 1:** Number of nodes showing statistically different intra- and inter-subject variabilities. In brackets: (number of nodes showing higher inter- vs. intra-subject variability/number of nodes showing higher intra- vs. inter-subject variability). The right column shows the average percentage of nodes (standard deviation in brackets), across metrics, where the inter-subject variability is significantly higher than the intra-subject one. The bottom row shows the percentage count of the first number in each cell.

Method	Betweenness Centrality	Local Efficiency	Strength	Clustering Coefficient	*Mean(sd) % out of 15*
PearsC	3(2/1)	4(0/4)	5(1/4)	3(0/3)	*5%(6%)*
PartC	7(3/4)	5(0/5)	7(2/5)	2(0/2)	8%(9%)
mGC	4(2/2)	5(2/3)	5(2/3)	15(15/0)	35%(38%)
mTE	3(1/2)	3(0/3)	4(1/3)	3(0/3)	*3%(3%)*
*Mean(sd) %* *out of 15*	*28%(13%)*	*28%(6%)*	*35%(8%)*	*38%(41%)*
